# “Do Health Messages Come from Mars or Venus?” The Effectiveness of Health Communication Depends on Gender Stereotypes in Messages

**DOI:** 10.3390/bs16060980

**Published:** 2026-06-12

**Authors:** Didier Courbet, Laure Jacquemier, Marie-Pierre Fourquet-Courbet, Esteban Courbet, Fabien Girandola

**Affiliations:** 1 Aix-Marseille University, Université de Toulon, IMSIC (Institut Méditerranéen des Sciences de l’Information et de la Communication), InCIAM (Institut Créativité et Innovations Aix-Marseille Université), 13005 Marseille, France; marie-pierre.fourquet@univ-amu.fr; 2 Aix Marseille University, CRET-LOG (Centre de Recherche sur le Transport et la Logistique), InCIAM (Institut Créativité et Innovations Aix-Marseille Université), 13625 Aix-en-Provence, France; laure.jacquemier@univ-amu.fr; 3 Department of Communication and Media Research, University of Fribourg, CH-1700 Fribourg, Switzerland; esteban.courbet@unifr.ch; 4 CARISM (Centre d’Analyse et de Recherche Interdisciplinaires sur les Médias), Paris-Panthéon-Assas University, 75006 Paris, France; 5 Aix Marseille University, LPS (Laboratoire de Psychologie Sociale), InCIAM (Institut Créativité et Innovations Aix-Marseille Université), 13621 Aix-en-Provence, France; fabien.girandola@univ-amu.fr

**Keywords:** persuasive communication, health communication, attitude change, preventive health, gender effect, social cognition

## Abstract

Prior research suggests that health messages can affect men and women differently, yet these differences and their underlying mechanisms remain insufficiently understood. Based on the premise that many health messages are implicitly gendered, this randomized controlled experiment (N = 1116), conducted in a high-risk real-world context, investigates the effectiveness of implicitly gendered messages on psychosocial determinants of protective behaviors, including cognitive, attitudinal, and motivational dimensions, as well as behavioral intentions. Twelve public health messages, derived from commonly used communications and theoretical frameworks, were first evaluated according to their perceived masculinity or femininity, and their effects were then experimentally tested across participants. Results indicate that messages strongly aligned with gender stereotypes produce the largest differences in effectiveness between men and women. For example, authority-based messages (a masculine stereotype) are more effective among men, whereas messages emphasizing social reciprocity or concern for others (feminine stereotypes) are more effective among women. These effects emerge only when recipients are likely to engage in systematic processing, particularly when their political stance diverges from that of the message source (the French government). The results support the gendered message–recipient gender congruence hypothesis, rather than alternative explanations based on gender-specific processing styles, with substantial practical implications.

## 1. Introduction

In many sectors, when ministries or public organizations seek to change behaviors among target populations, they frequently rely on persuasive communication campaigns. The objective is to disseminate messages to specific audiences through various media channels, including the Internet, television, radio, outdoor advertising, cinema, and the press. The aim is to create, reinforce, or modify cognitions, affects, motivations, attitudes, and behavioral intentions, with the ultimate goal of promoting the actual adoption of the targeted behaviors (Rice & Atkin, 2012).

Among communication recipients, several studies have highlighted gender differences in how messages are perceived and processed, for example, depending on the influence strategies used in the messages (R. Orji et al., 2015), or in more specific domains such as advertising (Brunel & Nelson, 2003; Wolin, 2003), politics (Reed, 2006; Schneider & Bos, 2023), and social issues (Eaton et al., 2017). Although the two main explanations for these differences rely on social role theory, they diverge in the cognitive processes involved, revealing a lack of theoretical clarity. First, each gender engages in different information-processing styles (Eaton et al., 2017). Compared to men, women tend to process messages more superficially, partly because they are socialized from a young age in Western societies to conform and preserve family harmony—this role encourages them to adjust their attitudes more readily (Eagly & Wood, 1991). In contrast, men are socially taught to change their opinions less. When exposed to persuasive messages, they adopt resistance-based processing, reducing their susceptibility to influence (Burgoon & Klingle, 1998).

The second explanation is message-centered: it posits that men and women respond differently depending on the content of each message. From this perspective, message effectiveness depends on the degree of congruence between gendered characteristics of the message and the recipient’s gender: a message containing feminine stereotypes (e.g., prosocial and relational messages) is more persuasive among women, whereas a message containing masculine stereotypes (e.g., authority or personal agency) is more effective among men (Carli, 2014). However, to our knowledge, from a theoretical standpoint, this explanation has rarely been empirically tested through systematic experimental designs.

There is one domain in which gender stereotypes strongly shape cognitions, attitudes, and behaviors: the health domain, as notably observed during the COVID-19 pandemic (Ferrín, 2022; Galasso et al., 2020). Beyond the important theoretical implications, the practical stakes are considerable because receiving communication messages that do not match individuals’ perceptions, attitudes, and behaviors may lead to absent effects, unexpected reactions, or even counterproductive outcomes (Ball & Wozniak, 2022). Indeed, delivering messages that correspond specifically to targeted audiences facilitates the adoption of protective behaviors.

To our knowledge, in the field of public health communication, no research has specifically examined gender effects explained by the degree of gendered message–recipient gender congruence. The present study aims, on the one hand, to demonstrate these effects and, on the other hand, to contribute to a better understanding of some underlying cognitive processes, particularly the type of processing involved. To this end, the study examines the interaction between gender effects and a fundamental concept in persuasion: the source of the messages, namely, as is often the case in public health contexts, the national government.

To advance beyond previous studies, this research incorporates several specific features. First, the design of the present study makes it possible to compare message effectiveness both within and between genders. This approach enables inter-gender comparisons (female vs. male participants), as well as intra-gender analyses by ranking the effectiveness of the twelve messages separately for each gender. At a practical level, this classification helps design more effective messages for target audiences.

Second, this experiment includes a greater number of messages than most prior studies (twelve), enhancing both its internal and external validity. These twelve messages use persuasion techniques grounded in theoretical frameworks, several of which are commonly employed in public health across many countries. They allow for an examination of twelve distinct levels of congruence with gender stereotypes. Each message reflects a specific degree of congruence with masculine and feminine stereotypes.

Third, this experiment offers high ecological validity, which is crucial in the context of public health. The study was conducted during the COVID-19 crisis, at a time when the risk to participants’ lives was real. Participants were drawn from the general population, and both the messages and the recommendation (“stay at home”) were realistic, with several drawn directly from official public health campaigns in various countries.

## 2. Theoretical Background

### 2.1. Gender and Message Influence

Gender differences in responses to persuasive messages (whether cognitive, attitudinal, or behavioral) are often explained through gender role theory (e.g., Wolin, 2003). Gender roles refer to the set of behaviors and social expectations associated with each sex (Eagly, 2013). These roles are largely shaped by gender stereotypes (beliefs about the attributes, skills, and behaviors considered appropriate for men and women in a given society; Hilton & Von Hippel, 1996).

These roles and stereotypes are internalized through socialization and become core components of both social and personal identity (Stoller, 1994). Two major hypotheses have been proposed to explain how gender influences persuasion. The first posits that men and women process persuasive messages differently (hereafter referred to as the “gender-driven message processing hypothesis”). Eaton et al. (2017) demonstrated that making gender roles salient through priming techniques affects how messages are received. When primed with feminine stereotypes, women showed greater susceptibility to persuasion due to weaker attitude strength and superficial message processing. Moreover, regardless of participants’ gender, feminine primes led to more superficial processing of messages. These processes would unfold automatically, meaning that they are difficult to control and require little to no cognitive resources (Bargh et al., 2012). Conversely, when primed with masculine stereotypes, both women and men engaged in more systematic and reflective message processing. According to Eaton et al. (2017), these findings may contribute to understanding why, on average, women are perceived as more persuadable than men (Wolin, 2003). These differences in message processing can be explained through gendered socialization processes. Women are often raised to conform more readily to social norms and to prioritize social harmony (Schneider & Bos, 2023). From an early age, they may learn to maintain peace within the family or social unit by adjusting their attitudes to reduce conflict (Eagly & Wood, 1991). Men, on the other hand, are socialized to more strongly defend their own viewpoints and to resist persuasion.

However, this first hypothesis has been challenged. Carli (2014) points out potential methodological biases in studies supporting this approach, which may distort the results. Theoretically, the model also presents limitations. Dual-process theories of persuasion (e.g., Petty & Cacioppo, 1986) suggest that the nature of the cognitive route, whether peripheral or central, does not, in itself, determine whether the outcome will be positive or negative. In other words, persuasion can result from both low- and high-effort processing, depending on context and content.

In public health communication campaigns, gender stereotypes are often present in messages, more or less implicitly, for example through the themes or arguments conveyed. These elements may signal that a message is more congruent with masculine or feminine norms, thereby shaping how recipients interpret and respond to it. Taken together, these perspectives suggest that recipients are more responsive both to the recommended behavior itself and to arguments featured in messages that are aligned with their own gender (Carli, 2014; R. Orji et al., 2015). Following the elaboration likelihood model (Petty & Cacioppo, 1986), a recipient’s motivation and ability to process a message depend on their interest in the topic and its perceived relevance. This, in turn, influences the degree of cognitive elaboration and the persuasive outcome. The present study is grounded in this theoretical framework, which constitutes the second explanatory hypothesis, according to which message effectiveness depends on the degree of gendered message–recipient gender congruence.

In Western societies, masculinity is often associated with agency (a focus on self-assertion, autonomy, and goal achievement) whereas femininity is associated with communality, or concern for others, cooperation, and emotional sensitivity (Eagly et al., 2020). Hentschel et al. (2019) further decompose these broad categories into several dimensions and subdimensions (see Table 1).

In general, women tend to show a stronger orientation toward prosocial behaviors (Abdullahi & Kumar, 2016) and express greater concern for both their own health and that of others (Carli, 2014; Courtenay, 2000). This makes them more responsive to messages that (1) promote health-related behaviors and (2) rely on prosocial appeals (e.g., helping others vs. helping oneself), as shown by Brunel and Nelson (2003) and R. Orji et al. (2015). Women are also more influenced by messages focusing on safety (Lewis et al., 2007; Rahn et al., 2021) and public health (Aldoory, 2001).

In most cases, a public health message includes at least two key components: (1) the recommended protective behavior to reduce risk (e.g., “To avoid catching and spreading COVID-19, you must stay at home”) and (2) the persuasive argument meant to motivate that behavior (Witte, 1992). These two elements are rarely studied separately in research, which can lead to interpretative ambiguities. For example, a single message might include a behavior aligned with feminine stereotypes and an argument aligned with masculine ones. In the present study, behaviors and arguments were separated to allow for a more precise investigation. The hypotheses tested posit that when either or both components (individually or taken together) are more aligned with masculine or feminine stereotypes, they will be more effective for the corresponding gender. Thus, the hypotheses derived from the “gendered message–recipient gender congruence hypothesis” are as follows:

**Hypothesis** **1.** *The Control message, which merely encourages compliance with the recommended preventive behavior without presenting any additional argument, as well as the full set of messages (combining behavior and persuasive argument), is more effective among female participants than among male participants.*

**Hypothesis** **2.** *Drawing on gender stereotype theory, within each gender group, the messages (combining behavior and persuasive arguments) differ in their effectiveness.*

In addition, one of the objectives is to establish a ranking of message effectiveness separately for female and male participants. Such rankings are essential for tailoring public health communication strategies to gender-specific target audiences.

We aim to test whether argument effectiveness depends on the degree of congruence between gendered arguments and recipients’ gender. Each persuasive argument is assumed to align to varying degrees with masculine or feminine stereotypes:

**Hypothesis** **3.** *We expect that the arguments most strongly differentiated along gender-stereotypical lines (i.e., highly masculine and minimally feminine, or vice versa) generate the largest differences in effectiveness between male and female recipients. For example, an argument that is both strongly masculine and weakly feminine should be especially effective among male participants.*

Based on these hypotheses, each of the 12 messages designed was associated with specific hypotheses. These are presented later in the manuscript, after detailing how each message was constructed and tested.

### 2.2. Understanding the Underlying Processes of Gender Effects

To go beyond existing studies in the literature, another objective of this experiment is to contribute to a better understanding of certain underlying cognitive processes, particularly the type of processing involved in gender effects in health communication.

Unlike explanations of gender effects that assume they depend on gender-specific processing (e.g., more superficial processing among women; Eaton et al., 2017), gender effects are instead conceptualized here as being driven by the gender stereotypes embedded in messages. To this end, the study examines the interaction between gender effects and a fundamental concept in persuasion: message source.

During major public health crises, important measures are announced by country’s political leaders. During the COVID-19 pandemic, in France as in many other countries, it was the head of state who served as the source of the messages indicating the necessity of lockdown. Even though heads of state often attempt to speak beyond political affiliations and to present themselves as defenders of the national interest, citizens frequently assess such messages through the lens of their political stance (Cakanlar & White, 2023). This was also observed during the COVID-19 pandemic (e.g., Sellnow-Richmond et al., 2023). In general, the degree of political agreement with the message source significantly affects how the message is evaluated and processed (Cakanlar & White, 2023). When recipients share the same political stance as the source, rather than an opposing one, they perceive the source as more credible, more competent, and more trustworthy. In this case, since source evaluation generally occurs before message processing, it influences the nature of that processing (Chaiken, 1980). Political stance congruence (vs. incongruence) triggers more superficial (vs. in-depth) message processing (Chaiken & Maheswaran, 1994; Druckman, 2001; Petersen et al., 2013). Individuals whose political stance diverges from that of the source engage in more in-depth processing, creating conditions under which congruence between gendered characteristics of the message and their gender becomes more influential.

In contrast, when recipients’ political stance aligns with that of the source, the cognitive resources allocated to message processing are much lower, if not nonexistent. As a result, stereotype processing, which requires cognitive resources, is disrupted, and the difference in effects between men and women disappears. However, according to the other explanatory hypothesis of gender effects, the gender-driven message processing hypothesis (Eaton et al., 2017), processing relies on automatic mechanisms that require little to no attention. In this situation, these mechanisms are still activated, and gender-related effects remain observable.

Moreover, when recipients share the same political stance as the source (versus an opposing one), it facilitates (versus hinders) persuasion (Avery, 2010) and, consequently, the adoption of recommended protective behaviors (Reynolds-Tylus, 2019; Wilson & Sherrell, 1993).

Based on these considerations and on the fact that the messages address themes related to community health and safety, which correspond to feminine stereotypes (Carli, 2014), Hypotheses 4 are formulated as follows. If the hypothesis of congruence between gendered messages and recipients’ gender holds, we expect to observe:

**Hypothesis** **4.** *Among individuals whose political stance does not align with that of the message source, gender effects are expected, due to more elaborative processing involving greater cognitive resources: message effectiveness is higher among women than among men. In addition, the variation in message effectiveness is greater among men than among women (H4.1). Among individuals whose political stance aligns with that of the message source, no gender effects are expected to emerge (H4.2), as message processing, and thus the processing of gender stereotypes, occurs with limited cognitive resources.*

## 3. Materials and Methods

### 3.1. Experimental Design and Messages Design

The study followed a one-factor between-subjects design with twelve conditions. Participants were randomly assigned to one of eleven experimental conditions or to a control condition, corresponding to twelve different persuasive messages. Each participant viewed only one message. Random assignment was verified. For the construction of the persuasive messages, five objectives were established. The messages had to:

(1) Be realistic, by using messages and arguments actually disseminated in public health, notably in COVID-19 prevention campaigns in different countries, in order to strengthen the ecological validity of the study.
(2) Be grounded in scientifically validated theories in order to achieve robust persuasive effectiveness (O’Keefe, 2015). The messages were developed based on theories of social influence (Cialdini, 2006), political communication (Condor et al., 2013), and public health communication. The theoretical concepts used to design the messages are detailed in Appendix A.
(3) Include diversified argumentative content to better examine persuasion effects and to cover a wide spectrum of gendered representations, ranging from weakly stereotyped messages to those explicitly mobilizing masculine or feminine stereotypes.
(4) Share the same structure and format to ensure that any differences in persuasive impact resulted from message content rather than from formal presentation features.
(5) Be easily understandable to the general public, ensuring accessibility and clarity for a wide audience.

Thus, the base message, used as a control condition, focused on health risk and included a behavioral recommendation: “Due to the COVID-19 pandemic, you must stay home.” Each of the 11 other messages included this same base message, followed by an additional argument inspired by a specific theoretical concept (see Table 2).

In France, President Emmanuel Macron announced the national lockdown in a public address on 13 March 2020. To ensure ecological validity, all messages in the experiment were attributed to Emmanuel Macron, who served as the stated source.

### 3.2. Double Manipulation Checks Through Five Pilot Tests

**Figure 1:**
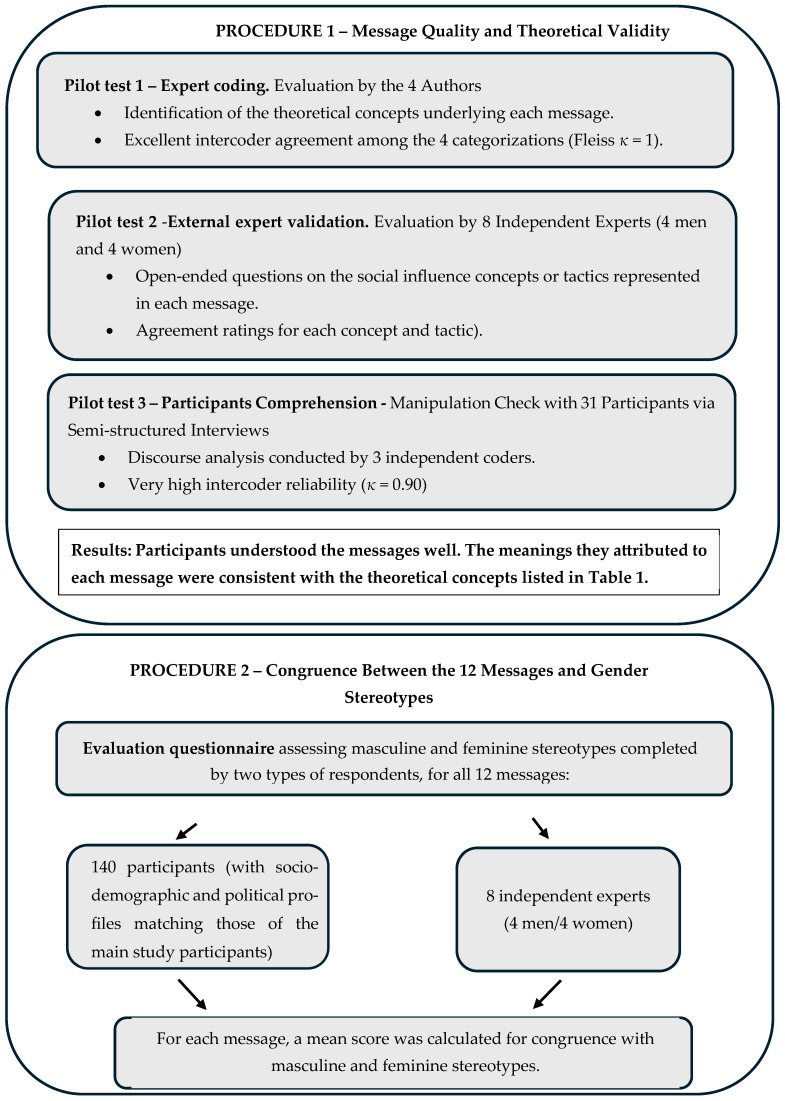
Dual manipulation check and pilot tests.

Preliminary trials integrating the manipulation checks into the main questionnaire revealed participant fatigue due to the length of the procedure. In accordance with methodological recommendations highlighting the potential drawbacks of embedded manipulation checks, the experimental manipulation was validated through independent pilot tests (Fayant et al., 2017; Hauser et al., 2018). The first manipulation check was carried out using three pilot tests to ensure that the messages were clearly understood and unambiguously grounded in theoretical concepts. The second manipulation check was conducted using two pilot tests to examine the relationship between the messages and gender stereotypes. Figure 1 summarizes the pilot tests and the dual manipulation check.

#### 3.2.1. First Manipulation Check

It involved three pilot tests conducted with distinct participant groups, following a triangulation-based validation approach (Denzin & Lincoln, 2011).

(1) Internal Expert Validation (Pilot test 1): In the first pilot test, the five authors of this study independently identified the main theoretical concepts or social influence tactics underlying each message. The level of agreement among their classifications was measured using Fleiss’ κ. The result indicated perfect agreement (κ = 1.0), reflecting complete inter-rater reliability (Landis & Koch, 1977).
(2) External Expert Validation (Pilot test 2): In the second pilot test, eight additional independent experts in persuasive communication (all tenured university professors and PhD-level researchers, 4 women and 4 men, all French nationals with no prior knowledge of the study’s objectives or design) individually completed a questionnaire. First, they responded to open-ended questions asking which theoretical concept or social influence tactic each message was based on, in their opinion. Then, for each concept previously identified during the first pilot test and listed in Table 2 (e.g., “This message is based on the concept of authority”), they rated their level of agreement using a 7-point Likert scale (1 = strongly disagree; 7 = strongly agree). Message order was randomized and responses were anonymized. Results from the open-ended responses showed near-perfect agreement among experts (*κ* = 0.85). No new concepts were proposed. Agreement with the proposed concepts was also high (*M* = 6.01; *SD* = 1.2).
(3) Validation with target audiences (Pilot test 3): The third pilot test aimed to assess the clarity and interpretability of the messages among target audiences. For this purpose, semi-structured interviews were the most appropriate method. Thirty-one participants whose profiles matched those of the experimental population were interviewed via videoconference between 3 and 8 April 2020, due to the lockdown. A detailed description of the sample and methodology is available elsewhere (Courbet et al., 2023). Participants answered open-ended questions to (1) assess their comprehension of the messages and (2) explore the meanings they attributed to each message. Three independent coders conducted a discourse analysis of the responses. Inter-coder reliability was excellent (κ = 0.90). The findings confirmed that participants’ interpretations of the messages matched the intended theoretical concepts listed in Table 2.

Consistent with the independent manipulation check procedures (Fayant et al., 2017; Hauser et al., 2018), the results of the three pilot tests demonstrated, on the one hand, that the messages were indeed grounded in the theoretical concepts they were intended to reflect and, on the other hand, that they were clearly understood by recipients. They provided strong evidence of construct validity.

#### 3.2.2. Second Manipulation Check

It was conducted to examine the relationship between the messages and gender stereotypes. Respondents completed a questionnaire in which they rated, on a 7-point scale, how well each message aligned with (1) masculine stereotypes and (2) feminine stereotypes, as defined in Table 1. Two types of respondents were surveyed:

(1) A sample of 140 individuals whose sociodemographic and political profiles were comparable to those of the experimental participants, recruited through the same social media channels;
(2) Eight independent experts (distinct from those in the first pilot test): 4 women and 4 men, all university-based scholar-researchers holding PhDs in social psychology or communication sciences. These experts had no prior knowledge of the study’s objectives or experimental design. In both cases, data collection was conducted online.

For each respondent type, *t*-tests were performed to compare means for each message and for the message set as a whole, where statistically appropriate. Further analyses identified the most strongly contrasted messages in terms of gender stereotypes. For the target audience, only effects with a large effect size (*d* > 0.80) were retained. The following four findings were observed consistently across (1) each of the two respondent types and (2) the average ratings across all two respondent types (see Table 2; Tables S1 and S2 in the Supplementary Materials):

(1) The preventive behavior requested in the *Control* message (without any argument) was more strongly associated with feminine stereotypes than with masculine stereotypes (e.g., among the target audience: *p* < 0.001, *d* = 0.57).
(2) Each message is more in line with a gender stereotype, either masculine or feminine.
(3) Overall, all constructed messages aligned more strongly with feminine than with masculine stereotypes (*p* = 0.02).
(4) The most stereotypically contrasted messages were: (a) *Authority* and *War* (more masculine); and (b) *Reciprocity* (more feminine). However, interviews conducted during the first pilot test revealed that the *War* argument, which had been used by the French President, was strongly rejected by both men and women. It was considered inappropriate for the COVID-19 context and therefore excluded from Hypothesis 3. Only *Authority* and *Reciprocity* were retained.

### 3.3. Participants

The experiment was conducted online between 3 April and 8 April 2020. A total of 1116 French adults aged 18 years or older participated. At the time of the study, all participants were under national lockdown in France, which had begun on 17 March 2020. Participants were recruited in two stages via social media platforms, primarily Facebook and LinkedIn. In the first stage, approximately thirty non-researchers with diverse profiles in terms of gender, age, occupation, and geographical location were asked to share, through social media and their extended online networks, a message indicating that participants were being sought for a study related to COVID-19. This message included a link to the online survey. No paid advertising was used, and the survey link was not posted in topic-specific groups. The sample can be described as follows: 68% identified as female and 32% as male. The mean age was 37.2 years (range: 18–86; *SD* = 17.4). Additionally, 14% lived alone, 50% lived with one or two other people; 58% had a garden, 28% had a balcony, and 14% had neither a garden nor a balcony. Ten percent were still working outside the home, while 58% were working from home. In terms of political stance, 19% were favorable toward the government, 26% were unfavorable, and 55% reported being neither favorable nor unfavorable.

### 3.4. Procedure

Participants first read an ethics statement and provided informed consent. They were then randomly assigned to view one of the 12 messages, each attributed to President Macron. After reading, they completed a questionnaire measuring message effectiveness, psychological responses, and demographic information. Item order was randomized to reduce order effects.

Ethical approval was obtained from InCIAM Aix-Marseille University (AMX-19-IET-005) and from the French National Research Agency (ANR-20-COV3-0003-01), which also reviewed the ethical aspects of the present project within the framework of an expedited COVID-19 emergency funding procedure, approved on 12 March 2020. The study was conducted in accordance with the ethical principles of the Declaration of Helsinki (World Medical Association, 2013) and the American Psychological Association (APA, 2017), and complied with the European General Data Protection Regulation (GDPR). All participants read a brief ethics statement, provided informed consent, and were informed of their rights, including data confidentiality and the voluntary nature of their participation.

### 3.5. Measures

To control the epidemic and promote public health in the context of lockdown, numerous behaviors had to be adopted. Therefore, an effective message had to optimally influence several distinct determinants of behavior involved in the psychosocial processes that extend from message reception to decision-making regarding protection-related behavioral intentions.

These behavioral determinants were identified across three complementary research domains: (1) studies on health behaviors (e.g., Ajzen, 1991); (2) studies on health behaviors in the context of COVID-19, including barrier gestures, and during lockdown, including nutrition-related behaviors (Chu et al., 2020); and (3) research on persuasive communication, which helps identify the stages of the persuasion process (Wagner & Petty, 2022).

Due to the unprecedented and unfamiliar nature of the disease and the lockdown measures, eleven distinct and complementary categories of determinants were identified, corresponding to eleven dependent variables, all considered equally important and covering cognitive, attitudinal, motivational, and behavioral dimensions.

All measures were theoretically grounded (see Section S1 in the Supplementary Materials) and grouped into four sets of variables. Responses were recorded on a scale ranging from 0 (“not at all”) to 10 (“absolutely”). Eleven outcome variables were assessed, grouped into four categories; all participants answered the eleven items after exposure to one randomly assigned message:

(1) Persuasive qualities of the message (Wagner & Petty, 2022):

- (1.1) The message’s ability to capture attention was measured using 3 items (“the message catches my attention,” “interests me,” “alerts me”; Cronbach’s *α* = 0.77).
- (1.2) Perceived personal relevance was assessed with the item: “The message is relevant to me.”
- (1.3) Cognitive evaluations of message credibility and persuasiveness were measured using 5 items (Cronbach’s *α* = 0.91): “The message is convincing,” “effective,” “credible,” “useful,” and “necessary.”
- (1.4) Attitude toward the message (“I appreciate the message”).

Measures (2), (3), and (4) assessed the effects of the messages on key determinants of health behavior:

(2) Motivation and perceived behavioral control (Ajzen, 1991; Chu et al., 2020)

- (2.1) Motivation to increase knowledge (2 items, Cronbach’s *α* = 0.85), including “The message encourages me to better understand the risks (the virus, the epidemic, its consequences for myself, my loved ones, and society)” and “to better understand the recommendations for self- and other-protection, such as preventive measures and lockdown guidelines.”
- (2.2) Effects on perceived behavioral control, specifically:
- (2.2.1) Motivation to protect oneself and others, assessed with 4 items (Cronbach’s *α* = 0.89): “The message motivates me to stay home,” “to correctly follow preventive measures,” “to protect the community,” and “to encourage my close contacts to do the same.”
- (2.2.2) Motivation to engage in healthy behaviors during lockdown, assessed with 4 items (Cronbach’s *α* = 0.88): “healthy eating,” “adequate sleep,” “physical activity,” and “avoiding harmful behaviors (e.g., alcohol, drugs, excessive screen time).”

(3) Measure (3) included two single-item measures focused on (Ajzen, 1991; Chu et al., 2020; Wagner & Petty, 2022):

- (3.1) perceived efficacy of the recommended action (“If I do not stay home, I might catch the virus”)
- (3.2) intention to share the message (particularly on social media).

(4) Measure (4) included two single-item measures assessing protective behavioral intentions (Ajzen, 1991; Chu et al., 2020; Wagner & Petty, 2022):

- (4.1) intention to follow recommended health behaviors (e.g., handwashing, distancing), and
- (4.2) intention to stay home.

Several reasons led to combining these variables into a single message effectiveness index, calculated as the mean of the eleven variables. This decision was theoretically grounded and made a priori. In the specific context of the COVID-19 lockdown, message effectiveness could not be reduced to one or two narrow dependent variables, such as message evaluation or the intention to adopt an isolated behavior. This choice reflects the theoretical objective of assessing the overall effectiveness of the messages across the full set of relevant determinants of protection. To be considered effective, a public health message had to influence several complementary determinants involved in a broader set of protection-related responses.

This complex protection process had to take into account, for example, the message’s ability to capture attention, its perceived personal relevance, its credibility, its perceived usefulness, motivation to learn more, motivation to protect oneself and others, motivation to adopt health-promoting behaviors during lockdown, the perceived effectiveness of the recommended action, the intention to share the message, and intentions to follow protective recommendations. These dimensions correspond to different stages and determinants of persuasion and health behavior identified in the three complementary theoretical domains that guided the construction of the measures, as described above. The eleven variables are equally important; it is the consideration of their entirety that gives meaning to the Message Effectiveness Index in the context of COVID-19. For these reasons, we retained the single Message Effectiveness Index as the main dependent variable. The index was therefore designed as a composite indicator of overall message effectiveness, rather than as a unidimensional psychometric scale in which all items would be expected to be interchangeable. Accordingly, the objective was not to establish an unidimensional scale, but to aggregate theoretically relevant indicators of message effectiveness. Thus, the Message Effectiveness Index benefits from theoretical validation.

The Message Effectiveness Index also benefits from empirical validation. After verifying the normality of each distribution and the homogeneity of variances for all eleven variables, we computed the overall Cronbach’s alpha, *α* = 0.86. Thus, this aggregation was also methodologically supported by the satisfactory internal consistency of the eleven variables. This good internal consistency suggests that the different measures, although referring to complementary components of message effectiveness, contributed sufficiently to the same global criterion to be combined into a single score. In this way, averaging the eleven variables made it possible to obtain a synthetic index of the overall protective effectiveness of the messages, in line with the theoretical objective of the study.

## 4. Results

For the overall analyses, multiple regression and analysis of variance (ANOVA) were used to examine both main effects and interaction effects of the key variables. All analyses were conducted using Statistica 13 software. In the detailed analyses, pairwise comparisons of message effectiveness scores (i.e., the average of the 11-item Message Effectiveness Index) were performed using *t*-tests. Prior to these comparisons, Levene’s test was used to assess the homogeneity of variances. One-tailed significance thresholds were applied when the direction of expected differences was specified in the hypotheses. Effect sizes were reported using *η*^2^ (partial eta squared) for ANOVAs and Cohen’s *d* for *t*-tests, with thresholds for small, medium, and large effects set at *η*^2^ = 0.01, 0.06, and 0.14, and *d* = 0.20, 0.50, and 0.80, respectively (Cohen, 1988). Additional regression-based moderation analyses were conducted using PROCESS Model 3 for SPSS version 31 (Hayes, 2022).

To predict message effectiveness, a multiple regression analysis was performed. Only variables with statistically significant results were included in the analysis: message type, gender, and political orientation (see Table 3). Regarding main effects, gender significantly influenced effectiveness: the messages were more effective among female participants than among male participants. Moreover, the *Control*, *Authority*, *Relatives*, *Self* + *Others*, *Reciprocity*, and *Lives* messages were more effective than the *Conformity* message.

**Figure 2:**
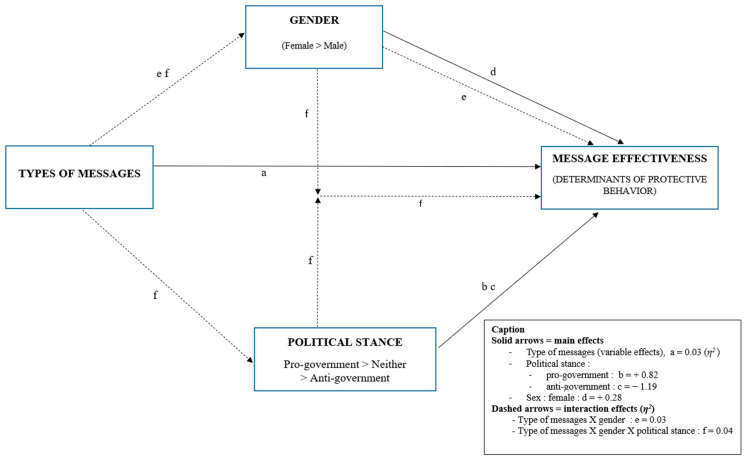
Overall results and relationships between variables.

Political orientation also influenced message evaluation: the messages were significantly less effective among participants who were politically opposed to the government. The messages were more effective among participants who were supportive of the government. Two types of interaction effects were also observed: (1) an interaction between message type and gender, and (2) a three-way interaction between message type, gender, and political orientation (see Figure 2). No significant effects were found for the variables age, number of household members, access to outdoor space (garden or balcony), or work location (home vs. outside the home).

### 4.1. Results for Hypotheses 1 and 2

**Figure 3:**
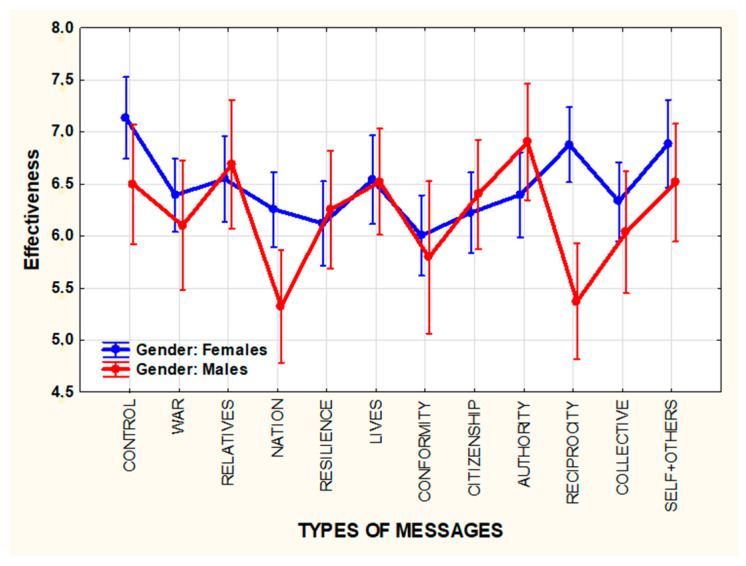
Effectiveness of the twelve messages according to gender.

The *Control* message was significantly more effective among female respondents (*M* = 7.13, *SD* = 1.44) than among male respondents (*M* = 6.50, *SD* = 1.48), *t*(89) = 1.98, *p* = 0.02, *d* = 0.43 (see Figure 3). The average effectiveness score across all 12 messages was significantly higher among female respondents (*M* = 6.47, *SD* = 1.54) than among male respondents (*M* = 6.21, *SD* = 1.75), *t*(1114) = 2.51, *p* = 0.008, *d* = 0.16. Hypothesis 1 was confirmed.

A one-way ANOVA was conducted separately for male and female participants to assess differences in message effectiveness across the 12 experimental conditions. Among women: *F*(11, 751) = 3.11, *p* = 0.0004, *η*^2^ = 0.04. Among men: *F*(11, 340) = 2.58, *p* = 0.0037, *η*^2^ = 0.08. These results confirm that message effectiveness varies within each gender. Furthermore, the variability in effectiveness was greater among male participants (*SD* = 1.75) than among female participants (*SD* = 1.54), Levene’s *F*(1, 1114) = 5.31, *p* = 0.02, lnSDR = 0.13, indicating a small-to-medium effect size. Hypothesis 2 was supported.

In Table 4, the messages were ranked according to their score on the effectiveness index. Consistent with the manipulation check results, among male participants, the most effective message was *Authority*. The two least effective messages were *Reciprocity* and *Nation*. Among female participants, *Control* was the most effective message, whereas *Conformity* was the least effective.

### 4.2. Results for Hypotheses 3 and 4

Regarding Hypothesis 3, which involved the *Reciprocity* and *Authority* arguments, a centering method was applied to specifically assess the relative effectiveness of these arguments, independently of overall gender differences in message effectiveness. The control message, which contained no argument, was excluded from the analyses. The ANOVA revealed a significant Gender × Message interaction, *F*(10, 1003) = 2.67, *p* = 0.003, *η*^2^ = 0.03..

For each gender, the effectiveness difference (Diff) between each argument and that gender’s average score across all arguments was calculated. Next, the difference score for each argument among men (diff M) and the difference score for each argument among women (diff W) were compared. The greatest gender-based differences were:

- *Reciprocity* (more effective among women): Diff_Females = 0.46 (*SD* = 1.55) vs. Diff_Males = −0.81 (*SD* = 2.05), *t*(45) = 3.11, *p* = 0.001, *d* = 0.93 (large effect).
- *Authority* (more effective among men): Diff_Males = 0.72 (*SD* = 1.56) vs. Diff_Females = −0.01 (*SD* = 1.44), *t*(87) = 2.22, *p* = 0.015, *d* = 0.53 (moderate effect). Hypothesis 3 was supported.

Regarding Hypotheses 4, overall message effectiveness was associated with participants’ political stance. The mean effectiveness score across the 12 messages was significantly higher among participants who were politically favorable toward the government (*M* = 7.36, *SD* = 1.25) than among those who were neither favorable nor unfavorable (*M* = 6.57, *SD* = 1.46), *t*(816) = 6.87, *p* < 0.001, *d* = 0.58. In turn, the neutral group’s mean was significantly higher than that of participants who were politically unfavorable (*M* = 5.34, *SD* = 1.57), *t*(909) = 11.57, *p* < 0.001, *d* = 0.81.

To further investigate gender effects within each political stance, separate Message × Gender ANOVAs were conducted. Among participants who were neither favorable nor unfavorable toward the government, a significant interaction between message type and gender was observed: *F*(11, 589) = 1.89, *p* < 0.05, *η*^2^ = 0.03. Overall, messages were more effective among female participants (*M* = 6.65, *SD* = 1.41) than among male participants (*M* = 6.35, *SD* = 1.57), *t*(611) = 2.28, *p* = 0.012, *d* = 0.21.

**Figure 4:**
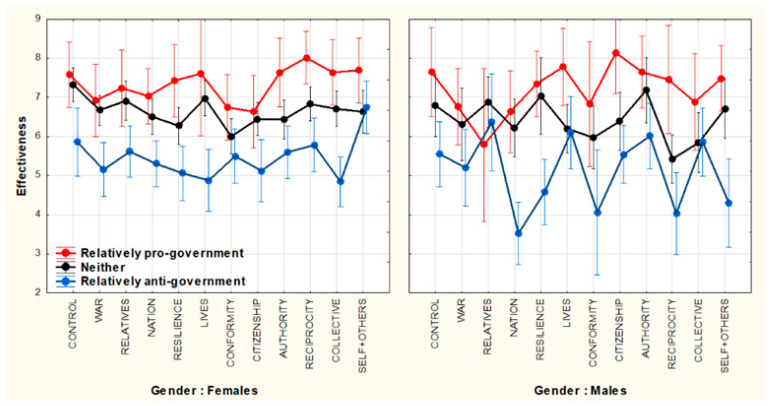
Effectiveness of the twelve messages according to gender and political stance.

Among participants who were politically unfavorable, the Message × Gender interaction was even stronger: *F*(11, 274) = 3.60, *p* = 0.0001, *η*^2^ = 0.13. Again, messages were rated as more effective by female participants (*M* = 5.46, *SD* = 1.50) than by male participants (*M* = 5.14, *SD* = 1.69), *t*(296) = 1.64, *p* < 0.05, *d* = 0.20. Standard deviations also differed significantly, being larger among male respondents: *F*(1, 296) = 3.7, *p* < 0.05. This difference in variability was not found in the other political stance groups, suggesting that, overall, message effectiveness was more variable among male participants than female participants (see Figure 4).

Among politically unfavorable participants, gender significantly influenced the effectiveness of four specific messages, each with a large effect size. *Self + Others* (*t*(21) = 3.72, *p* = 0.001, *d* = 1.62), *Nation* (*t*(32) = 4.21, *p* = 0.00027, *d* = 1.53) and *Reciprocity* (*t*(21) = 2.58, *p* = 0.017, *d* = 1.13) were significantly more effective among women than among men. Conversely, the *Lives* message was more effective among male respondents than among female respondents (*t*(19) = 2.23, *p* = 0.037, *d* = 1.02).

Among participants favorable toward the government, no significant effects were found. Neither the overall message effectiveness scores nor comparisons by gender revealed any significant differences. These findings are consistent with Hypotheses 4.1 and 4.2.

Additional 2 × 2 × 2 regression-based moderation analyses were conducted using PROCESS Model 3 to further examine the gendered message–recipient gender congruence hypothesis. The twelve experimental conditions were recoded into two categories based on the manipulation check results: messages containing masculine stereotypes and messages containing feminine stereotypes. This variable was entered as the independent variable (X). Participant gender was entered as the first moderator (W). Political stance was also recoded as congruent versus non-congruent with the source, with participants favorable toward the government coded as congruent and those who were either unfavorable or neither favorable nor unfavorable coded as non-congruent. This variable was entered as the second moderator (Z). Message effectiveness was entered as the dependent variable (Y).

The overall model was significant, *F*(7, 1108) = 17.46, *p* < 0.001, *R*^2^ = 0.10. The Message Gender Stereotype × Participant Gender interaction was significant, *b* = 0.67, *SE* = 0.24, *t* = 2.76, *p* = 0.006, indicating that the relative effectiveness of masculine- versus feminine-stereotyped messages differed according to participant gender.

With respect to Hypotheses 4.1 and 4.2, the three-way interaction involving political stance congruence with the source was in the expected direction but did not reach conventional levels of statistical significance, *b* = −0.84, *SE* = 0.53, *t* = −1.59, *p* = 0.112. One possible explanation concerns the use of PROCESS Model 3, a regression-based moderation approach which, in the present case, led us to simplify the original structure of the data by recoding the twelve message conditions into binary dummy-coded variables (Hayes, 2022).

This global pattern remained unchanged when the control message was excluded, as this message did not contain an additional persuasive argument. The overall model was significant, *F*(7, 1017) = 16.50, *p* < 0.001, *R*^2^ = 0.10. The Message Gender Stereotype × Participant Gender interaction remained significant, *b* = 0.64, *SE* = 0.25, *t* = 2.58, *p* = 0.010. The three-way interaction was again in the expected direction but non-significant, *b* = −0.80, *SE* = 0.54, *t* = −1.50, *p* = 0.130.

Taken together, these additional analyses provide convergent support for the gendered message–recipient gender congruence hypothesis.

## 5. Discussion

Conducted in an epidemic context, this randomized controlled experiment aimed to demonstrate that the effectiveness of public health messages depends on the degree of congruence between the gender stereotypes, sometimes implicitly conveyed in message content, and the gender of the recipients. The research also aimed to contribute to a better understanding of the cognitive processes underlying these effects by examining interactions with source effects in persuasion. To our knowledge, this experiment is the first to demonstrate this effect, and it does so with a high level of ecological validity. This validity stems from the real-life context of a public health crisis involving serious and immediate risks. This congruence between gendered messages and recipients’ gender was observed at three levels: the recommended protective behavior, the arguments promoting that behavior, and the messages considered as a whole. The large number of messages tested (twelve), using influence techniques commonly employed in public health, all grounded in theoretical frameworks, further enhances the internal and external validity of the study, particularly for both inter-gender and intra-gender comparisons. This interpretation extends a broader framework recently proposed by Albarracin et al. (2024). Messages whose content is congruent with a salient identity of the recipient are processed more fluently and feel more compelling, whereas identity-incongruent messages tend to elicit resistance or resentment. Our findings transpose this identity-congruence logic to gender identity in the public health domain, where it had not yet been systematically tested.

### 5.1. Gender-Based Differences in Message Effectiveness, Consistent with Gender Stereotypes

Given that women are generally more health-conscious, the *Control* message (which recommends preventive behavior without any additional argument) was more effective among female recipients than among male recipients, with a medium effect size (Hypothesis 1). This result is consistent with previous research showing that compared to men, women are more aware of health risks, experience more negative emotions related to those risks, and are quicker and more likely to adopt protective behaviors (Ferrín, 2022; Galasso et al., 2020). Also consistent with gender stereotype theory, the set of messages that combined a behavior and a persuasive argument was generally more effective among female participants than male participants, though with a small effect size (Hypothesis 1). Furthermore, message effectiveness varied within each gender group (Hypothesis 2), with effect sizes ranging from medium to large. Male participants’ responses were more varied across message types than those of female participants, leading to more pronounced differences in message effectiveness among males.

### 5.2. Gendered Argument Effectiveness, Consistent with Gender Stereotypes

*Reciprocity* (more effective among female respondents) and *Authority* (more effective among male respondents) were the most strongly gendered arguments according to data from the pilot test, and also produced the largest gender differences in effectiveness.

Among female recipients, *Reciprocity* was particularly effective (it ranked second out of twelve in this group) and it was significantly less effective among male recipients (ranked 11th out of 12). These findings are consistent with R. Orji et al. (2015) for female participants, but diverge for male participants, for whom *Reciprocity* had previously been found to be more persuasive.

One result is worth highlighting. Of the thirteen conclusions from the pilot tests on stereotype-message congruence (see Table 2), eleven were confirmed by the experimental data. This indicates that the pilot tests correctly anticipated eleven out of thirteen outcomes. A binomial test showed that this level of confirmation was statistically significant (*p* = 0.01), underscoring the heuristic value of further investigating gender effects in persuasion. These results contribute to the literature by experimentally showing that, in public health communication, message influences on men and women depend on the degree of gendered message–recipient gender congruence. Baxter et al. (2022), in a UK study on walking-promotion leaflets, refine this point. The negative effect of communal (feminine) wording emerges almost exclusively among men with a strong masculine gender role identity, while other men and all women are largely insensitive to it. This pattern helps explain the greater across-message variability observed here among male recipients and suggests that gender role identity—rather than biological sex alone—may act as the key moderator.

### 5.3. Gender Effects Would Require Processing with Substantial Cognitive Resources

Hypotheses 4 broadened the level of observation of gender effects in order to better understand the nature of the processes involved during message reception. The results show that the gendered message–recipient gender congruence hypothesis is compatible with research focusing on another fundamental persuasion variable: the source.

As a preliminary remark, we first note that message effectiveness varies according to participants’ political stance: it is higher among those who are favorable to the government, intermediate among individuals who are neither favorable nor unfavorable, and lower among those who are unfavorable. The effect sizes can be considered moderate to large, indicating that political stance notably influences the extent to which the messages achieve their intended effects. These results, consistent with the existing literature (Chaiken, 1980), suggest that source evaluation processes were likely activated by participants and contribute to validating the experimental procedure.

The main series of statistical analyses suggests that when the source’s political stance was not congruent with that of the recipients, gender effects emerged: message effectiveness was higher among women than among men. Moreover, given that the arguments varied from one message to another, variation in message effectiveness was greater among men in this incongruent condition. In contrast, when the source’s political stance was congruent with that of the recipients, no gender effect was observed. Additional statistical analyses pointed in the same direction, although they did not reach statistical significance, suggesting that further research with larger samples is needed.

Persuasion research suggests that political agreement with the source tends to promote superficial message processing (Chaiken, 1980), whereas disagreement encourages more elaborated content processing (Druckman, 2001; Petersen et al., 2013). Accordingly, the results of this study are consistent with the predictions derived from the gendered message–recipient gender congruence hypothesis. Gender effects seem to appear primarily when recipients are likely to engage in deeper message processing, namely, when their political stance diverges from that of the source. Under these conditions, recipients may have been more likely to scrutinize the gender stereotypes conveyed by the message. The results provide stronger support for the congruence hypothesis between gendered messages and the recipients’ gender than for the gender-driven message processing hypothesis (Eaton et al., 2017). According to this latter hypothesis, gender differences in persuasion result from automatic processing. Therefore, regardless of whether recipients agreed or disagreed politically with the source, gender effects should have emerged, since automatic processing requires little or no cognitive resources.

Further experimental research more directly targeting cognitive processes is nevertheless needed to confirm this explanation. It would be particularly useful to determine whether the effect observed here is mainly driven by elaborated processing alone, as suggested by dual-process models of persuasion (Chaiken, 1980; Petty & Cacioppo, 1986), or whether it is accompanied by a complementary process of motivated skepticism or bias correction toward the source (Taber & Lodge, 2006; Wegener & Petty, 1995). In this framework, bias correction refers to recipients’ attempts to neutralize the perceived influence of a source they consider potentially biased or lacking credibility, by assigning less weight to that source in their judgment. In this latter case, political incongruence with the source may first activate greater vigilance toward the source, or even an attempt to distance oneself from its influence, which could then lead recipients to process message content more deeply and become more sensitive to its gendered features. Future research should therefore examine whether source-related vigilance, correction of the source’s perceived influence, and elaborated message processing operate independently, sequentially, or jointly in the emergence of gender effects in persuasive health communication.

Several more specific findings also emerge. Gender was also a significant predictor of effectiveness for four messages, all with large effect sizes. Specifically, the *Self + Others*, *Reciprocity*, and *Nation* messages were significantly more effective among female participants, as expected based on stereotype-congruence pilot tests. In contrast, the *Lives* message was more effective among male participants. Although this last finding was not predicted, two possible explanations may account for it: (1) it could reflect the activation of gendered cognitions specific to the COVID-19 context, or (2) it may indicate an ongoing shift in gender stereotypes in France, which have evolved in recent years (Eagly et al., 2020; Hentschel et al., 2019). Moreover, even during a health crisis, when the head of state aims to rise above differences in political stance, citizens tend to evaluate messages through the lens of their own political stance. These results are consistent with other findings from the COVID-19 pandemic (Warren & Lofstedt, 2022; Sellnow-Richmond et al., 2023).

### 5.4. Message Effectiveness by Gender

Message rankings were established separately for female/male participants, enabling intra-gender analyses and allowing identification of the most and least effective messages for each group. These findings are useful for adapting public health communication strategies by selecting arguments that best resonate with specific gender-based audiences. Gender stereotype theory effectively explains several aspects of message processing: overall message effectiveness, the impact of the *Control* message (which contains only the behavior recommendation), and the most pronounced gender-based argument effects. However, some results suggest that factors beyond gender stereotypes may also shape how messages are processed, opening new avenues for future research.

#### 5.4.1. Message Effectiveness Among Women

First, the most effective message among female participants was the *Control* message, which presented the recommended preventive behavior without any additional argument. This aligns with the notion that feminine stereotypes emphasize health-related behaviors. Through socialization, women internalize these norms, making them more likely to adopt the recommended behavior even in the absence of persuasion. Adding arguments slightly reduced the message’s effectiveness. This finding is consistent with prior studies showing that prosocial messages (Favero & Pedersen, 2020; Jordan et al., 2021) or theoretically framed persuasive appeals (Pink et al., 2023) are not necessarily more effective than simple, straightforward messages. This pattern is reinforced by the recent meta-analysis of Grimani et al. (2026) on prosocial public-health messaging to reduce respiratory infection risk: across studies, prosocial appeals yield only modest and inconsistent effects relative to simpler informational messages, echoing our observation that adding arguments to the simple recommendation tended to weaken its impact on women.

Although some pairwise comparisons reached the conventional threshold of *p* < 0.05, they should be interpreted with caution because of the large number of Student’s t-tests conducted. After Bonferroni correction, only effects reaching the more stringent threshold of *p* < 0.001 should be considered statistically robust. Therefore, results that do not reach this corrected threshold should not be interpreted as confirmatory evidence, but rather as exploratory trends. Nevertheless, they open up promising avenues for future research on the differential effectiveness of messages.

Second, the processing of some messages appeared to involve information beyond what is explained by gender stereotypes. For example, *Self + Others* ranked second in effectiveness among female participants, despite balancing self-interest and concern for others (a framing less stereotypically feminine). Similarly, the *Authority* message, which was rated as not particularly feminine in the pilot tests, ranked in the middle.

Third, *Conformity* was the least effective message among female participants. This finding also applied to men and is consistent with R. Orji et al. (2015), although it contradicts Cialdini’s (2006) hypothesis that conformity-based appeals should be effective during periods of uncertainty. While previous research suggests women are generally more responsive to conformity appeals (R. O. Orji et al., 2013), this was not supported in the present study.

Fourth, the *Collective* and *Nation* messages (ranked 2nd and 4th in gender stereotype congruence during pretesting) were among the least effective for female participants. One explanation may be that women, like men, also consider dominant cultural norms, such as a preference for individualism and a stronger identification with micro-groups over macro-level entities (e.g., nation or society). As such, macro-social arguments invoking national unity, collective resilience (Condor et al., 2013), or group conformity (Asch, 1955; Cialdini, 2006) were less persuasive than micro-social appeals centered on direct surroundings or personal impact (e.g., “I save lives”).

#### 5.4.2. Message Effectiveness Among Men

As expected, based on masculine stereotypes, the *Authority* message was the most effective among male participants. Conversely, the *Reciprocity* message ranked lowest (tied with another). The relatively low effectiveness of the *Nation* message may also reflect the influence of dominant cultural themes in how these messages were processed. Interestingly, the *Relatives* message, ranked second among male participants, appeared to have been evaluated based on considerations beyond traditional masculine stereotypes. Like female respondents, male respondents also expressed a desire to protect their close relatives. During the third pilot test of the manipulation check, male respondents reported fearing more for their loved ones than for themselves—a finding that diverges from traditional masculine norms and aligns with results from Bokemper et al. (2022) and Christner et al. (2020), who found this argument effective across genders.

Finally, the salience of the *Authority* appeal for male recipients can be read in light of Dallimore et al. (2024). Using a serial-reproduction paradigm, the authors explain that, within a society, gender stereotypes spontaneously re-emerge through social transmission, beginning in childhood, even when the initial information is gender-balanced. This suggests that authority-based influence among men reflects stable cultural structures that are unlikely to shift rapidly, despite documented evolutions in stereotype content (Eagly et al., 2020; Hentschel et al., 2019).

### 5.5. Limitations, Practical Implications and Research Perspectives

Nonetheless, the study has certain limitations. First, the results apply specifically to the COVID-19 pandemic context and to the sampled population. Second, the measures focused on behavioral determinants rather than on actual behaviors.

Third, the sample included a higher proportion of women than men. This imbalance does not directly affect the analyses conducted separately among women and men, since responses from both groups were not aggregated into an overall mean. However, estimates for men were based on a smaller subsample and may therefore be less precise. Direct gender comparisons may also have had lower statistical power. Accordingly, gender differences should be replicated in samples with a more balanced gender distribution. Fourth, despite the large number of participants in the experiment and the diversity of their profiles, the sample was not representative of the French population. For example, older adults without access to social media platforms could not be surveyed. Fifth, the exceptional historical context of the COVID-19 crisis may have heightened emotional responses and, in turn, influenced the causal relationships, compared with what would be observed in a more ordinary public health context.

From a practical standpoint, this study underscores the importance of tailoring persuasive arguments to the recipient’s gender, taking into account associated gender stereotypes. Such adaptations may help optimize public health communication strategies and prevent potentially counterproductive effects for a given gender group. However, when mass communication channels such as television or radio are used, it may be difficult to target men and women separately with gender-specific messages. When both genders are exposed to the same messages, the experimental results show that the most effective message is the simplest, non-argumentative one: “Due to the pandemic, you must stay at home.” Overall, adding arguments reduces its effectiveness. The next two most effective messages emphasized that adopting the recommended protective behavior was framed (a) as a way “to protect yourself and others” and (b) as being “for the good of your family, friends, and people close to you.”

Future research should further examine how stereotypes and other cognitions interact in gender-differentiated message processing, particularly as gender stereotypes evolve over time (Eagly et al., 2020; Hentschel et al., 2019). Similarly, the gendered message–recipient gender congruence hypothesis should be examined in relation to other persuasive communication variables related to the recipient, the message, the medium, or the source. To assess the degree to which messages are gendered, the use of a quantified indicator would be preferable to a simple nominal male–female categorization, as it may increase the likelihood of experimentally detecting relationships with other variables. It would also be valuable to extend this work to other countries to enable cross-cultural comparisons rooted in differing gender stereotypes. As this study focuses on the health domain, where gender stereotypes tend to be predominantly feminine, further studies should investigate behavior change processes associated with more masculine stereotypes. Regarding the socio-cognitive processes involved, beyond the more specific examination of the possible role of a complementary process of motivated skepticism or bias correction toward the source (Wegener & Petty, 1995), already discussed above, another avenue for future research could be pursued: integrating the two complementary explanations of gender differences in message effectiveness, namely the gendered message–recipient gender congruence hypothesis and the gender-driven message processing hypothesis (Eaton et al., 2017), would provide a more comprehensive understanding of these effects.

## 6. Conclusions

This randomized controlled experiment, conducted in the context of a real-life and life-threatening crisis, helps us better understand gender effects in public health communication, as well as the underlying cognitive mechanisms that may explain them. In everyday life, a large number of public health messages contain gender stereotypes, either explicitly or implicitly. The results highlight the central role of the degree of congruence between the gender stereotypes conveyed by the messages and the recipients’ gender in explaining message effectiveness. The theoretical perspective developed in this experiment could also be studied and applied in other domains of persuasive communication beyond public health. Indeed, the gendered message–recipient gender congruence hypothesis may help explain the effects of persuasive messages aimed at changing behaviors in a variety of contexts, including prosocial domains such as environmental protection or social inclusion, as well as commercial advertising.

## Figures and Tables

**Table 1:** Dimension scales and scale items (inspired by Hentschel et al., 2019).

Agency Dimensions	Communality Dimensions
**1. Instrumental Competence**	**1. Concern for Others**
Competent	Understanding
Effective	Kind
Productive	Compassionate
Task-Oriented	Sympathetic
**2. Leadership Competence**	**2. Sociability**
Leadership Ability	Communicative
Achievement-Oriented	Collaborative
Skilled In Business Matters	Relationship-oriented
**3. Assertiveness**	Likeable
Dominant	**3. Emotional Sensitivity**
Bold	Emotional
Assertive	Intuitive
Competitive	Sentimental
**4. Independence**	
Independent	
Desires Responsibility	
Emotionally Stable	
Self-Reliant	

**Table 2:** Experimental messages, associated concepts and associated gender stereotypes (for further details, see Figure 1; Appendix A; Supplementary Materials: Tables S1 and S2).

Conditions	Messages	Social Influence Tactics and Associated Concepts	Message Congruence with Masculine (Ma) and Feminine (Fe) Stereotypes Following the Manipulation Check			
			M Fe *	M Ma *	Diff M (M Fe − M Ma *)	Gender stereotypes
*Authority*	CCM ** + We categorically ask you to respect this measure	Authority (Cialdini, 2006)	1.98	5.47	−3.49	Masculine
*War*	CCM ** + We are at war with the virus	Political rhetoric of war (Condor et al., 2013)	2.85	5.05	−2.2	Masculine
*Resilience*	CCM ** + We will come out of this crisis stronger	Resilience and growth of the country (Condor et al., 2013)	3.28	4.73	−1.45	Masculine
*Relatives*	CCM ** + For the good of your family, friends and people close to you	Protecting attachment figures (Bowlby, 1969)	3.99	4.02	−0.03	Masculine
*Lives*	CCM ** + By doing so, you are saving lives	High level of action identification (Vallacher & Wegner, 2014) and social valuation	3.98	3.57	0.41	Feminine
*Citizenship*	CCM ** + Let’s act for the common good	Unit 1: citizenship (Cialdini, 2006)	4.67	3.43	1.24	Feminine
*Nation*	CCM ** + The nation is with you and will be grateful to you	National social support and gratitude (Condor et al., 2013)	4.77	3.47	1.3	Feminine
*Self + Others*	CCM ** + To protect yourself and others	Double values: Self/others (Triandis & Gelfand, 1998)	4.82	3.12	1.7	Feminine
*Control condition*	Due to the COVID-19 pandemic, you must stay at home (CCM **)		4.75	2.99	1.76	Feminine
*Conformity*	CCM ** + Behave like everyone else	Conformity (Asch, 1955); Social proof (Cialdini, 2006)	4.86	2.98	1.88	Feminine
*Collective*	CCM ** + We are all united, together in the same situation	Unit 2: common destiny, belonging to a united collective (Cialdini, 2006)	5.21	2.75	2.46	Feminine
*Reciprocity*	CCM ** + Healthcare workers are helping you, please help them	Reciprocity (Cialdini, 2006)	5.31	2.48	2.83	Feminine
All messages			4.19	3.65	0.54	Feminine

*Notes.* * M = mean of the two respondent types to the questionnaire; Ma = Masculine; Fe = Feminine. ** CCM = control condition message.

**Table 3:** Main effects (multiple regression predicting message effectiveness) and interaction effects of the tested variables.

	**Main Effects**					
**Variables**	N *	**Coefficient (B)**	**Standard Error (SE)**	***t***	***p***	**Significant**
Type of messages **						
Intercept *(Conformity)*	82	5.87	0.18	32.65	<0.001	Yes
*Control*	91	0.99	0.22	4.50	<0.001	Yes
*Citizenship*	99	0.42	0.21	1.96	0.049	Yes
*Authority*	89	0.71	0.22	3.24	0.001	Yes
*Relatives*	82	0.75	0.22	3.36	0.001	Yes
*Resilience*	89	0.28	0.22	1.27	0.203	No
*War*	103	0.37	0.21	1.76	0.078	No (trend-level effect)
*Nation*	107	0.11	0.21	0.51	0.607	No
*Self + Others*	84	0.81	0.22	3.61	<0.001	Yes
*Reciprocity*	105	0.45	0.21	2.14	0.032	Yes
*Collective*	95	0.40	0.22	1.82	0.069	No (trend-level effect)
*Lives*	90	0.67	0.22	3.04	0.002	Yes
**Gender** (females)	759	0.28	0.09	3.03	0.002	Yes
**Political Stance** (anti-government)	290	−1.19	0.10	−11.65	<0.001	Yes
**Political Stance** (pro-government)	212	0.82	0.12	7.01	<0.001	Yes
	**Interaction Effects**					
**Variables**	**ANOVA; *p***			**Significant**		**Effect Size**
Type of messages × Gender (global)	*F*(11,1044) = 2.48, *p* = 0.004			Yes		*η*^2^ = 0.03
Type of messages × Gender × Political Stance (global)	*F*(22,1044) = 1.99, *p* = 0.004			Yes		*η*^2^ = 0.04

*Note*. * N refers to the number of participants in each category: participants exposed to each message condition, or participants in each gender or political-stance subgroup. ** The ANOVA confirms the results by showing main effects of the variable “type of messages”: *F*(11, 1044) = 2.92, *p* < 0.001, *η*^2^ = 0.03. Effect sizes are reported as partial eta squared (*η*^2^).

**Table 4:** Message Effectiveness Index among female and male respondents.

Females (N = 764)				Males (N = 352)			
Message	Mean (SE) for Females	95% CI		Message	Mean (SE) for Males	95% CI	
		*LL*	*UL*			*LL*	*UL*
*Control*	7.13 (0.20)	6.74	7.53	*Authority*	6.90 (0.28)	6.35	7.46
*Self + Others*	6.89 (0.22)	6.46	7.31	*Relatives*	6.69 (0.32)	6.07	7.31
*Reciprocity*	6.88 (0.18)	6.52	7.24	*Lives*	6.52 (0.26)	6.01	7.03
*Relatives*	6.55 (0.21)	6.14	6.96	*Self + Others*	6.51 (0.29)	5.95	7.08
*Lives*	6.54 (0.22)	6.11	6.97	*Control*	6.50 (0.29)	5.92	7.07
*Authority*	6.40 (0.21)	5.99	6.8	*Citizenship*	6.40 (0.27)	5.88	6.93
*War*	6.39 (0.18)	6.04	6.74	*Resilience*	6.25 (0.29)	5.69	6.82
*Collective*	6.33 (0.19)	5.95	6.71	*War*	6.10 (0.32)	5.48	6.72
*Nation*	6.26 (0.18)	5.90	6.62	*Collective*	6.04 (0.30)	5.45	6.62
*Citizenship*	6.22 (0.20)	5.84	6.61	*Conformity*	5.80 (0.37)	5.06	6.53
*Resilience*	6.12 (0.21)	5.72	6.53	*Reciprocity*	5.37 (0.28)	4.81	5.93
*Conformity*	6.01 (0.20)	5.62	6.39	*Nation*	5.32 (0.28)	4.78	5.86

*Note.* Messages are ranked in descending order according to their mean effectiveness score, separately for female and male respondents. Means, standard errors (SE), and 95% confidence intervals (CI) are reported as descriptive statistics. LL = lower limit; UL = upper limit. Additional statistical analyses of differences in message effectiveness are provided in the Supplementary Materials, Table S3a,b.

## Data Availability

The authors are willing to share their data and analytics methods with other researchers. The material is available upon request.

## Supplementary Materials

The following supporting information can be downloaded at https://www.mdpi.com/article/10.3390/bs16060980/s1. Supplementary Material S1: Section S1. Theories Underlying Experimental Measurements. Supplementary Material S2: Table S1. Pretest Results: Expert Ratings (n = 8) on Congruence with Gender Stereotype. Supplementary Material S3: Table S2. Pretest Results on Gender Stereotype Congruence, General Public Sample (n = 140). Supplementary Material S4: Table S3a. Pairwise Student’s *t*-Tests Comparing Message Effectiveness Among Male Participants: t Values (df). Supplementary Material S5: Table S3b. Pairwise Student’s *t*-Tests Comparing Message Effectiveness Among Female Participants: t Values (df).

## Appendix A. Theories Used to Design the Messages

Eleven theoretical concepts from three research domains were used. Although some concepts from these three domains have been tested in the context of the COVID-19 epidemic—and even used in prevention campaigns in some countries—none had been examined in relation to gender stereotypes and the respondent’s gender.

(1) Cialdini (2006) identified several social influence tactics frequently used in various types of media communication. These refer specifically to:

(1.1) Social proof: In situations of uncertainty, social proof reflects the tendency to behave or legitimize one’s behavior based on others’ behavior or judgment. This tactic relies on social norms and conformity, a powerful factor in social influence (Asch, 1955), and has proven effective in the context of COVID-19 (Bokemper et al., 2022);

(1.2) Authority: This encourages obedience in certain situations;

(1.3) Reciprocity: Doing a favor for someone implies that something may be expected in return, thus activating the social norm of reciprocity. In the context of COVID-19, Pink et al. (2023) observed small but significant effects of such a message focused on caregivers;

(1.4) Unity: This refers to the shared identity between the influencer and the influenced person. In the context of COVID-19, two sub-tactics can be distinguished. The first (1.4.1) appeals to civic motivation. The second (1.4.2) appeals to the feeling of belonging to a united community and shared destiny. Kelly and Hornik (2016) showed that messages framed in terms of benefits for society were more effective than those focused on the self.

(2) Three political communication techniques were tested (see Condor et al., 2013).

(2.1) When the French President Emmanuel Macron announced the population lockdown, he declared: “We are at war against the virus.” This statement uses a political communication technique derived from war rhetoric, designating a common enemy to prepare for a collective, almost physical, “fight” (see Condor et al., 2013).

(2.2) The provision of national social support based on nationalist values;

(2.3) Highlighting national resilience by promising a positive future and greater development of the country’s strength after the crisis.

(3) Three concepts from public health communication:

(3.1) Messages that call for the protection of close others, particularly those to whom we are emotionally attached (Bowlby, 1969) as attachment figures, are crucial for everyone. Some research results appear counterintuitive. For example, Capraro and Barcelo (2020) found that messages encouraging people to protect their family had no effect on mask-wearing to prevent COVID-19 transmission, whereas messages encouraging people to protect their community did have an effect;

(3.2) Messages that valorize each individual by showing that protective behaviors are identified as higher-level actions, attributing to them more human and prosocial meanings. Indeed, according to Vallacher and Wegner (2014), the same action can be identified at multiple levels by the person performing it: it can carry different meanings across different levels and contexts. An action may be identified at a low level (e.g., acting for short-term operational reasons, like protecting oneself to avoid catching COVID-19) or at a high level (e.g., for socially respected values and longer-term goals, such as saving human lives);

(3.3) Messages based on a call to protect, and which rely on values of individualism (i.e., self-protection) and collectivism (i.e., protecting oneself and others). While these two values may seem opposed, Triandis and Gelfand (1998) suggest that individuals may be motivated both by valuing themselves and by valuing others. In the context of COVID-19, some studies have shown that both motivations predicted social distancing behavior (Fazio et al., 2021), others found that motivation to protect others was a stronger predictor than motivation to protect oneself (Christner et al., 2020), and still others found that appeals to care for others were no more effective than the informative control message (Favero & Pedersen, 2020).
