# Supplementary material for: “Do Health Messages Come from Mars or Venus?” The Effectiveness of Health Communication Depends on Gender Stereotypes in Messages
Source: Behav Sci (Basel). 2026 Jun 12;16(6):980. doi: 10.3390/bs16060980 (PMC13296204; doi:10.3390/bs16060980)
Supplement: Supplementary file 1 [file behavsci-16-00980-s001.zip › Supplementary_Material_S1_SectionS1.pdf]

Supplementary Material S1.

Section S1. Theories Underlying Experimental Measurements

The measures are grounded in three complementary scientific fields (hereafter referred to as A, B, and C) that elucidate the characteristics that an effective public health message must possess to act effectively. The numbers below are identical to the measure numbers in the text.

Firstly, concerning its cognitive and affective characteristics, the literature on persuasion (A) (Wagner & Petty, 2011) indicates that the message must (1.1) sufficiently attract attention, alert, and interest the audience; (1.2) be considered personally relevant; (1.3) be considered credible and convincing; and (1.4) at the affective level, be favorably evaluated (i.e., elicit a positive attitude).

Secondly, with regard to the motivational and behavioral characteristics of the message in the context of the COVID-19 pandemic (C) (Chu et al., 2020), the literature on health behavior change (B) indicates that it must act on the following determinants of behavior (Ajzen, 1991; Matsunaga et al., 2023):

(2.1) The message must encourage awareness of risks and their consequences as well as awareness of risk-avoidance recommendations (Witte, 1992);

(2.2) An effective message acts on perceived behavioral control (Ajzen, 1991), and more specifically on motivation to protect oneself and others, such as the motivation

(2.2.1) to stay at home, properly practice barrier measures, and encourage family and friends to do the same, on the one hand, and

(2.2.2) the motivation to adopt good physical health practices during lockdown (e.g., healthy diet, sleep, physical activity, and avoiding harmful behaviors) on the other.

An effective message (3.1) raises awareness of the effectiveness of recommendations (Rogers & Prentice-Dunn, 1997) and (3.2) increases the intention to spread them to others (Gough et al., 2017). It also increases intentions to act and implement recommendations, such as (4.1) practicing barrier measures and (4.2) observing lockdown restrictions.
