# Supplementary material for: “Do Health Messages Come from Mars or Venus?” The Effectiveness of Health Communication Depends on Gender Stereotypes in Messages
Source: Behav Sci (Basel). 2026 Jun 12;16(6):980. doi: 10.3390/bs16060980 (PMC13296204; doi:10.3390/bs16060980)
Supplement: Supplementary file 1 [file behavsci-16-00980-s001.zip › Supplementary_Material_S2_TableS1.pdf]

Supplementary Material S2.

Table S1. Pretest results: expert ratings (n = 8) on congruence with gender stereotypes

Messages Conditions	Congruence with Feminine Stereotypes $M_{(F)}$*	Congruence with Masculine Stereotypes $M_{(M)}$*	Diff $M_{(F)} - M_{(M)}$*	Conclusion
Authority	1.88	5.75	-3.88	Masculine
War	3.13	5.25	-2.13	Masculine
Resilience	3.13	5.13	-2.00	Masculine
Relatives	4.63	4.75	-0.13	Masculine
Lives	3.13	2.50	0.63	Feminine
Citizenship	4.75	3.25	1.50	Feminine
Nation	4.75	3.13	1.63	Feminine
Self+Others	4.88	2.63	2.25	Feminine
Control condition	4.75	2.38	2.38	Feminine
Conformity	5.25	2.88	2.38	Feminine
Collective	5.63	2.25	3.38	Feminine
Reciprocity	5.50	2.00	3.50	Feminine
**All messages**	**4.28**	**3.49**	**0.79**	**Feminine**

Notes. *$M_{(F)}$ = Mean rating for congruence with feminine stereotypes; $M_{(M)}$ = Mean rating for congruence with masculine stereotypes. Higher positive differences indicate stronger congruence with feminine stereotypes; negative differences indicate stronger alignment with masculine stereotypes.
