# Supplementary material for: “Do Health Messages Come from Mars or Venus?” The Effectiveness of Health Communication Depends on Gender Stereotypes in Messages
Source: Behav Sci (Basel). 2026 Jun 12;16(6):980. doi: 10.3390/bs16060980 (PMC13296204; doi:10.3390/bs16060980)
Supplement: Supplementary file 1 [file behavsci-16-00980-s001.zip › Supplementary_Material_S3_TableS2.pdf]

Supplementary Material S3.

Table S2. Pretest results on gender stereotype congruence, general public sample (n = 140)

Messages Conditions	Agreement with feminine stereotypes M(F)*	SD (Feminine)	Agreement with masculine stereotypes M(M)*	SD (Masculine)	ddl	t	p (one-tailed)	Cohen's d	Conclusion
Authority	2.07	1.36	5.18	1.72	260	16.69	< .001	2.07	Masculine
War	2.57	1.76	4.86	2.19	260	9.55	< .001	1.18	Masculine
Resilience	3.40	1.76	4.33	1.67	275	4.54	< .001	0.55	Masculine
Relatives	3.364	1.97	3.29	2.08	273	0.30	.383	0.03	Feminine (NS)
Lives	4.827	1.57	4.64	2.17	247	0.79	.216	0.09	Feminine (NS)
Citizenship	4.59	1.87	3.62	1.84	275	4.39	< .001	0.53	Feminine
Nation	4.78	1.81	3.81	1.63	272	4.69	< .001	0.56	Feminine
Self+Others	4.75	1.85	3.61	2.11	270	4.79	< .001	0.58	Feminine
Control	4.76	1.99	3.60	1.96	272	4.86	< .001	0.59	Feminine
Conformity	4.474	2.05	3.09	1.74	263	6.01	< .001	0.73	Feminine
Collective	4.79	1.99	3.25	1.90	271	6.54	< .001	0.79	Feminine
Reciprocity	5.12	1.99	2.95	1.72	268	9.72	< .001	1.17	Feminine
**All messages**	**4.10**	**1.20**	**3.82**	**1.16**	**278.00**	**1.97**	**.02**	**0.24**	**Feminine**

Note. **M(F)* = Mean rating for congruence with feminine stereotypes; *M(M)* = Mean rating for congruence with masculine stereotypes; NS = not significant.
