# Supplementary material for: “Do Health Messages Come from Mars or Venus?” The Effectiveness of Health Communication Depends on Gender Stereotypes in Messages
Source: Behav Sci (Basel). 2026 Jun 12;16(6):980. doi: 10.3390/bs16060980 (PMC13296204; doi:10.3390/bs16060980)
Supplement: Supplementary file 1 [file behavsci-16-00980-s001.zip › Supplementary_Material_S4_TableS3a.pdf]

Supplementary Material S4.

Table S3a. Pairwise student's t-tests comparing message effectiveness among male participants: t values (df)

	CONDITIONS	1 Control	2	3	4	5	6	7	8	9	10	11	12
2	*War*		—										
3	*Relatives*			—									
4	*Nation*	2.51 (61)*		3.22 (56)**	—								
5	*Resilience*					—							
6	*Lives*				2.82 (68)**		—						
7	*Conformity*			2.11 (41)*				—					
8	*Citizenship*				2.43 (66)*				—				
9	*Authority*				**3.57 (62)*** **			2.27 (47)*		—			
10	*Reciprocity*	2.42 (58)*		2.96 (54)**			2.59 (66)*		2.22 (64)*	3.31 (60)**	—		
11	*Collective*									2.07 (57)*		—	
12	*Self+Others*				2.51 (61)*						2.3 (59)*		—

Notes. Empty cell = not significant. *$p < .05$. **$p < .01$. ***$p < .001$. Pairwise comparisons were conducted between the 12 experimental message conditions using independent-samples t-tests. Each message effectiveness score was compared with the effectiveness score of each of the other messages. In light of the number of pairwise comparisons performed, a Bonferroni-type correction would require a more stringent significance threshold than the conventional .05 level. In the table, t-values corresponding to comparisons significant at p < .001 are shown in bold to identify the most robust differences.
