# Supplementary material for: “Do Health Messages Come from Mars or Venus?” The Effectiveness of Health Communication Depends on Gender Stereotypes in Messages
Source: Behav Sci (Basel). 2026 Jun 12;16(6):980. doi: 10.3390/bs16060980 (PMC13296204; doi:10.3390/bs16060980)
Supplement: Supplementary file 1 [file behavsci-16-00980-s001.zip › Supplementary_Material_S4_TableS3b.pdf]

Supplementary Material S5.

Table S3b. Pairwise student's t-tests comparing message effectiveness among female participants: t values (df)

	**CONDITIONS**	**1 Control**	**2**	**3**	**4**	**5**	**6**	**7**	**8**	**9**	**10**	**11**	**12**
2	*War*	2.82 (138)**	—										
3	*Relatives*	2.18 (117)*		—									
4	*Nation*	**3.5 (134)*** **			—								
5	*Resilience*	**3.76 (119)*** **				—							
6	*Lives*	2.06 (113)*					—						
7	*Conformity*	4.46 (124)**		2.06 (119)*				—					
8	*Citizenship*	3.3 (124)**							—				
9	*Authority*	2.8 (118) **								—			
10	*Reciprocity*				2.5 (146)*	2.8 (131)**		3.43 (136)**	2.39 (136)*		—		
11	*Collective*	2.94 (127)**									2.02 (139)*	—	
12	*Self+Others*				2.58 (126)*	2.93 (111)*		**3.62 (116)*** **	2.45 (116)*			2.07 (119)*	—

Note. Empty cell = not significant. **p* < .05. ***p* < .01. ****p* < .001. Pairwise comparisons were conducted between the 12 experimental message conditions using independent-samples t-tests. Each message effectiveness score was compared with the effectiveness score of each of the other messages. In light of the number of pairwise comparisons performed, a Bonferroni-type correction would require a more stringent significance threshold than the conventional .05 level. In the table, t-values corresponding to comparisons significant at p < .001 are shown in bold to identify the most robust differences.
